# Prognostic models for outcome prediction in patients with advanced hepatocellular carcinoma treated by systemic therapy: a systematic review and critical appraisal

**DOI:** 10.1186/s12885-022-09841-5

**Published:** 2022-07-09

**Authors:** Li Li, Xiaomi Li, Wendong Li, Xiaoyan Ding, Yongchao Zhang, Jinglong Chen, Wei Li

**Affiliations:** Department of Cancer Center, Beijing Ditan Hospital, Capital Medical University, 100015 Beijing, China

**Keywords:** Hepatocellular carcinoma, Systemic treatment, Prognostic models, Review and critical appraisal

## Abstract

**Objective:**

To describe and analyze the predictive models of the prognosis of patients with hepatocellular carcinoma (HCC) undergoing systemic treatment.

**Design:**

Systematic review.

**Data sources:**

PubMed and Embase until December 2020 and manually searched references from eligible articles.

**Eligibility criteria for study selection:**

The development, validation, or updating of prognostic models of patients with HCC after systemic treatment.

**Results:**

The systematic search yielded 42 eligible articles: 28 articles described the development of 28 prognostic models of patients with HCC treated with systemic therapy, and 14 articles described the external validation of 32 existing prognostic models of patients with HCC undergoing systemic treatment. Among the 28 prognostic models, six were developed based on genes, of which five were expressed in full equations; the other 22 prognostic models were developed based on common clinical factors. Of the 28 prognostic models, 11 were validated both internally and externally, nine were validated only internally, two were validated only externally, and the remaining six models did not undergo any type of validation. Among the 28 prognostic models, the most common systemic treatment was sorafenib (*n* = 19); the most prevalent endpoint was overall survival (*n* = 28); and the most commonly used predictors were alpha-fetoprotein (*n* = 15), bilirubin (*n* = 8), albumin (*n* = 8), Child–Pugh score (*n* = 8), extrahepatic metastasis (*n* = 7), and tumor size (*n* = 7). Further, among 32 externally validated prognostic models, 12 were externally validated > 3 times.

**Conclusions:**

This study describes and analyzes the prognostic models developed and validated for patients with HCC who have undergone systemic treatment. The results show that there are some methodological flaws in the model development process, and that external validation is rarely performed. Future research should focus on validating and updating existing models, and evaluating the effects of these models in clinical practice.

**Systematic review registration:**

PROSPERO CRD42020200187.

**Supplementary Information:**

The online version contains supplementary material available at 10.1186/s12885-022-09841-5.

## Background

Hepatocellular carcinoma (HCC) is an important public health problem, ranking sixth in incidence and third in mortality globally [1]. The World Health Organization (WHO) estimates that more than 1 million people will die from HCC in 2030, which will impose a serious economic and emotional burden on people around the world [2]. One of the main reasons for the poor prognosis of patients with HCC is that they have entered the intermediate and late disease stages when diagnosed [3]. Typically, the standard treatment for advanced HCC is systemic treatment, wherein great progress has been made in recent years. Targeted therapy drugs including sorafenib, lenvatinib, regorafenib, cabozantinib, and ramucirumab; checkpoint inhibitors such as nivolumab and pembrolizumab; combinations such as atezolizumab-bevacizumab, and other systemic therapy drugs, including FOLFOX-4, have been applied in clinical practice.

HCC are highly heterogeneous. Therefore, patient stratification based on prognosis would optimize the choice of treatment and confer more benefits. At present, a variety of staging systems have been developed to evaluate the prognosis of patients with HCC, such as the American Joint Committee on Cancer (AJCC) tumor-node-metastasis (TNM) staging system [4], the Barcelona Clinic Liver Cancer (BCLC) staging system [5], the Cancer of the Liver Italian Program (CLIP) score [6], the Okuda staging system [7], the Japan Integrated Staging (JIS) score [8], and the Chinese University Prognostic Index (CUPI) [9]. However, whether these staging systems are applicable to patients with HCC receiving systemic treatment has not been systematically described and analyzed.

Although great progress has been made the treatment of advanced HCC, the overall prognosis of HCC after treatment remains poor. Therefore, standardized selection of treatment methods is particularly important, and the emergence of prognosis models can help solve this problem. Alpha-fetoprotein (AFP) has always been considered the most important prognostic indicator of HCC. In addition, many clinical indicators are closely related to HCC prognosis. Multivariate prognostic models developed with these clinical indicators evaluate the prognosis of HCC to classify patients to provide the best treatment, while reducing the burden on patients and the medical system.

At present, many multivariable prognostic models predicting the clinical outcome of patients with HCC treated with systemic therapy have been developed, but whether their predictions are reliable is unclear. Therefore, we summarized and analyzed these predictive models.

## Methods

We designed this systematic review and critical appraisal according to systematic review and meta-analysis of prediction model performance [10] and Checklist for critical Appraisal and data extraction for systematic Reviews of prediction Modelling Studies (CHARMS) [11], and guided by Li Wei and Chen Jinglong. A proposal for the study was published on PROSPERO (registration number CRD42020200187).

### Literature search

We systematically searched PubMed and Embase from the beginning of the database to 31 December 2020 to gain all studies developing and/or validating a prognostic model for all clinical outcomes in HCC patients who have received systemic treatment. We created the following search strategy:((hepatocellular OR Hepatic OR Liver) AND (carcinom* OR Cancer OR Neoplasm* OR Malign* OR Tumor) OR (Hepatocellular Carcinoma) OR (Liver Neoplasms)) AND (Systematic therapy OR immunotherapy OR targeted therapy OR Sorafenib OR Lenvatinib OR Regorafenib OR Nivolumab OR Pembrolizumab OR Camrelizmab OR Cabozantinib OR Ramucirumab OR FOLFOX-4) AND (Predict* OR Progn* OR Risk prediction OR Risk score OR Risk calculation OR Risk assessment OR C statistic OR Discrimination OR Calibration OR AUC OR Area under the curve OR Area under the receiver operator characteristic curve OR Nomogram). Two researchers (LiLi, Li Xiaomi) independently did the literature search, and a third researcher (Li Wei) resolved the discrepancies. In addition, we searched the references of eligible articles to find other potential additional eligible articles.

### Eligibility criteria

We included all studies that reported the development and/or validation of predictive models for all clinical outcomes of HCC patients who have received systemic treatment. Table S1 detailed the PICOTS of this review [10, 11]. We followed the Transparent Reporting of a multivariable prediction model for Individual Prognosis Or Diagnosis (TRIPOD) statement to select eligible prognostic model studies [12]. These studies were the development, validation and update of prognostic models for individualized predictions of HCC patients with systemic therapy. The selected objects were HCC patients who undergone systemic treatment. The patients have been diagnosed as HCC through histological biopsy or imaging examination. The systemic treatment drugs include sorafenib, lenvatinib, regorafenib, cabozantinib and ramucirumab, nivolumab, penbrolizumab, FOLFOX-4 and other systematic treatments. The selected clinical outcomes should include any possible clinical endpoints. Among HCC patients, the most common outcome indicators are overall survival (OS) and progression-free survival (PFS). Predictors of prognostic models are readily available and have been proven to be associated with prognosis of the patients. The studies of external validation of the existing models require systemic therapy to HCC patients, and the model’s performance was estimated [13].

We excluded diagnostic models that developed or validated to predict HCC, and prognostic models developed for HCC patients receiving other treatments (liver resection, liver transplantation, ablation and transarterial chemoembolization, etc.). In addition, we also excluded cross-sectional studies because the predictors and clinical outcomes were measured concurrently, which is not a predictive study.

### Data extraction

We constructed a form according to the CHARMS checklist [11], and standardized extraction of data for each article. In the articles that developed models, we extracted the following information: first author, publication year, model name, country, intervention, validation type, sample size, clinical outcome, predictors, C statistic, 95% confidence Interval (CI), the presence of Receiver operating characteristic (ROC) curve and calibration chart. There are many indicators for evaluating model performance. In order to facilitate statistics, we have extracted the C statistic as the discrimination measure, and the calibration plot as the potential calibration measure. When the same predictive model has multiple clinical outcomes, we retained the clinical outcome of the main analysis in the study. When the same predictive model performs prognostic analysis in the overall population and specific subgroups of the population, we retained the analysis of the overall population. From article describing external validation models, we extracted the following information: model name, C statistic and 95% CI, clinical outcome, validation type, sample size, first author and publication year.

### Risk of bias assessment

We evaluated the risk of bias in the development of prognostic model research by using the Prediction model Risk Of Bias Assessment Tool (PROBAST), which is a risk of bias assessment tool designed for systematic reviews of diagnostic or prognostic prediction models [14–16]. It contains four different domains: participants, predictors, outcomes and statistical analysis. According to the characteristics of the research, the answer to the question is yes, probably yes, no, probably no and no information. If a domain contains at least one question indicated as “no” or “probably no”, it is graded as high risk. If all the questions contained in a domain are answered with “yes” or “probably yes”, the domain is grades as low risk. When all domains are low risk, the overall risk of bias is considered to be at low risk; when at least one domain is high risk, the overall risk of bias is considered to be in high risk. Two researchers (Li Li, Xiaomi Li) independently assessed the risk of bias. We summarized the characteristics of the models based on descriptive statistics, calculated the median range of continuous variables, and the respective percentages of binary variables.

### Patient and public involvement

No patients participated in the formulation of research questions or outcome measures, nor did they participate in the formulation of research design or implementation plans. The patients were not asked to make suggestions for the recording and interpretation of the results. There are no plans to disseminate the results of the study to study participants or the relevant community of patients.

## Results

**Fig. 1:**
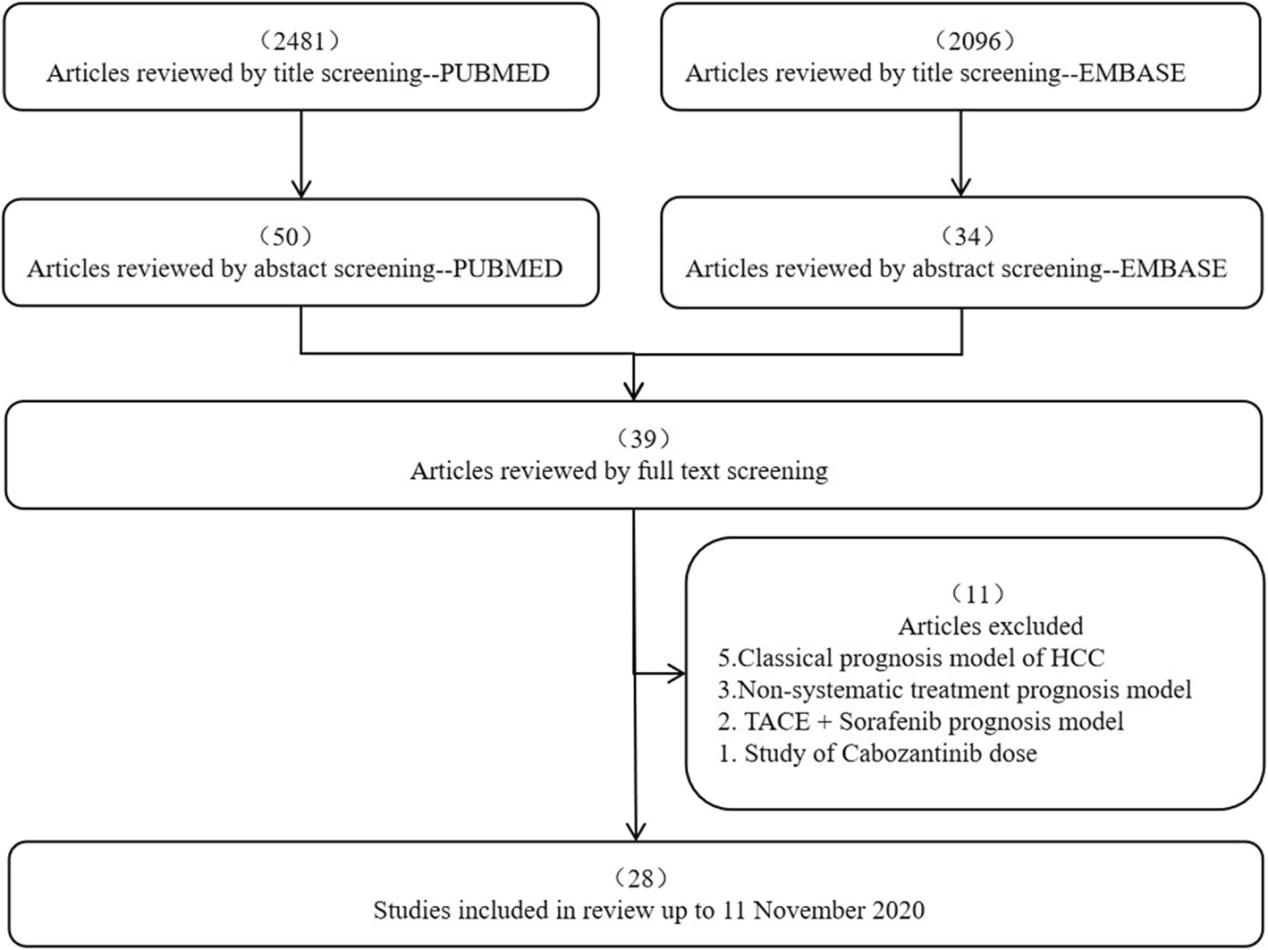
Flowchart of literature search for prognostic models in patients with hepatocellular carcinoma

Forty-four eligible articles were screened from PubMed and Embase, the search flow was shown in Fig. 1. Among them, 28 articles described the development of 28 prognostic models for patients with HCC after systemic treatment (details shown in Table 1), and 16 articles described the external validation of 32 existing HCC prognostic models [17–32]. Among the 32 externally validated prognostic models, 12 were externally validated > 3 times, and the C statistics (with 95% CI) or the number of events (in this case, the death cases) were reported.

**Table 1:** Overview of prediction models for diagnosis and prognosis of HCC

Author,year	Model	Country	Intervention	Validation type	Sample size(N)	Outcome	Predictors	AUC (95%CI)	Discrimination plot	Calibration plot
Adhoute X, 2016 [33]	NIACE	France	Surgery, TACE, Sorafenib	IV,EV	161	OS	the number of nodules, the infiltrating nature of the HCC, AFP, CP, ECOG PS	0.784	Absent	Absent
Berhane S, 2019 [17]	PROSASH	UK	sorafenib	IV	588	OS	vascular invasion, age, ECOG PS, AFP, albumin, creatinine, AST, extra hepatic spread and aetiology	0.72 to 0.70	Absent	Present
Chan SL, 2019 [34]	ILIS	Hong Kong Newcastle	sorafenib	IV,EV	627	OS	albumin, Bilirubin, alkaline phosphatase, Neutrophil, AFP	/	Absent	Absent
Choi GH, 2014 [35]	SPSM	US	sorafenib	IV,EV	356	OS	CP, AFP, tumor morphology, vascular invasion, extrahepatic involvement	0.809 (0.765–0.868)	Present	Present
Conroy G, 2017 [36]	IBSs-SII	France	sorafenib	/	161	OS	AST, BMI, SII, CP-B, macroscopic vascular invasion	/	Absent	Absent
Di Costanzo GG,2015 [37]	3-GPI	Italy	sorafenib	IV,EV	226	OS	AFP, bilirubin, ALT	/	Absent	Absent
Di Costanzo GG, 2017 [38]	OTE	Italy	sorafenib	IV	279	OS	Skin toxicity, diarrhoea, arterial hypertension	0.715 (0.645–0.785)	Present	Absent
Diaz-Beveridge R, 2017 [39]	La Fa	Spain	sorafenib	IV	145	OS	ECOG PS, CP score, early onset diarrhoea, bNLR	0.69	Absent	Absent
Edeline J, 2017 [40]	SAP	France, UK	sorafenib	IV,EV	370	OS	ECOG PS, AFP, tumour size, bilirubin, albumin	0.732 (0.669–0.789)	Absent	Absent
Ha Y, 2020 [41]	LMR-N	Korea, US	sorafenib	/	297	OS	LMR, treatment location, previous treatment, PS, AFP, lymph node metastasis, CP score	0.71 (0.67–0.75)	Absent	Absent
Howell J, 2017 [42]	RD-CLIP	Japan, Italy and UK	sorafenib	/	442	OS	CLIP score, RDW, treatment-related diarrhoea	0.808 (0.734–0.882)	Absent	Absent
Kim HY,2018 [43]	NBBM	Korea	sorafenib	IV	124	OS	etiology, platelet count, BCLC, PIVKA- II, HGF, FGF	0.825 (0.734–0.915)	Present	Absent
Kinoshita A,2013 [44]	GPS	Japan	TACE, TAI, systemic chemotherapy, sorafenib or BSC	/	150	OS	AST, total bilirubin, decreased albumin, AFP, larger tumor diameter, tumor number, vascular invasion, extra hepatic metastasis, CP, CLIP	0.897 (0.699–0.876)	Present	Absent
Labeur TA,2020 [18]	PROSASH-II	Bordeaux, France, Germany, Amsterdam, Rotterdam	sorafenib	IV,EV	615	OS	the serum albumin, bilirubin, AFP, macrovascular invasion, extrahepatic spread, largest tumour size	0.63 (0.60–0.66)	Absent	Absent
Lee HW,2017 [45]	NEXT	Korea	sorafenib	IV,EV	272	OS	ECOG PS, CP score, serum sodium, AFP	0.809 (0.758–0.860)	Present	Absent
Nakanishi H,2016 [46]	BCP	Japan	sorafenib	/	165	OS	CRP, albumin, AFP, lack of major vascular invasion	/	Absent	Absent
Pan QZ,2015 [47]	ACIKCI-N	China	adjuvant CIK cell immunotherapy	IV	1031	OS	tumor size, tumor capsule, pathological grades, total bilirubin, albumin, PT, AFP, tumor number	0.698 (0.677–0.719)	Present	Present
Qin S,2017 [48]	FOLFOX4-N	mainland China, Taiwan, Korea, and Thailand	FOLFOX 4	IV	184	OS	age, maximum tumor diameter, lymph node status, AST, TBIL, AFP	0.75 (0.71–0.80)	Absent	Present
Sprinzl MF,2018 [49]	GPS-EP	Germany	sorafenib	IV	120	OS	ECOG PS, PVTT, GPS	0.826 (0.746–0.907)	Present	Absent
Takeda H,2015 [19]	JRC	Japan	sorafenib	IV	270	OS	distant metastases, PVTT, intrahepatic tumor burden, AFP, des-γ-carboxyprothrombin, albumin, total bilirubin	0.755 (0.707–0.803)	Absent	Absent
Tang C,2020 [50]	9-MRG	①TCGA②ICGC	sorafenib	EV	374	OS	RRM2, DTYMK, LPCAT1, LCAT, TXNRD1, G6PD, PTGES, ENTPD2, UCK2	0.797	Present	Present
Yoo JJ,2017 [20]	SCHCC	Korea	sorafenib	IV	612	OS	CP, AFP, tumor type, extrahepatic metastasis, PVTT	0.818	Absent	Absent
Yuan J,2017 [51]	PBNC-N	China	sorafenib	/	464	OS	HBsAg, neutrophil count, thrombus, metastasis, tumor size	0.79	Present	Present
Liu T,2020 [52]	7-IRGBM	TCGA, ICGC	/	IV,EV	374	OS	BIRC5, FOS, DKK1, FGF13, IL11, IL17D, SPP1	0.778	Present	Absent
Huo J,2020 [53]	IGPPM	TCGA, ICGC	/	IV,EV	374	OS	45 immune-gene pairs with general applicability	0.899	Present	Absent
Xu D,2020 [54]	8-IPSHCC	TCGA, ICGC	/	IV,EV	423	OS	CKLF, IL12A, CCL20, PRELID1, FYN, GLMN, ACVR2A, CD7	0.79	Present	Absent
Wang WJ,2020 [55]	10-IRGPM	China, TCGA	/	IV,EV	374	OS	BIRC5, CSPG5, IL-11, FABP6, FIGNL2, GAL, IL17D, MAPT, SPP1, STC2	0.818	Present	Absent
Wang Z,2020 [56]	9-NIRPM	TCGA,ICGC	/	IV,EV	337	OS	ANGPT1, MAPT, DCK, SEMA3F, IL17D, HSPA4, RBP2, NDRG1, OSGIN1	0.811	Present	Absent

*IV* Internal validation, *EV* External validation, *OS* Overall survival, *HCC* Hepatocellular carcinoma, *AFP* Alpha-fetoprotein, *CP* Child Pugh score, *ECOG PS* Eastern Cooperative Oncology Group score standard, *AST* Aspartate aminotransferase, *BMI* Body mass index, *SII* Systemic immune inflammation index, *NLR* Neutrophil to lymphocyte ratio, *LMR* Lymphocyte-to-monocyte ratio, *CLIP* Cancer of Liver Italian Program, *RDW* Red cell distribution width, *PIVKA- II* Protein Induced by Vitamin K Absence or Antagonist-II, *HGF* Hepatocyte growth factor, *FGF* Fibroblast growth factor, *CRP* C-reactive protein, *PT* Prothrombin time, *PVTT* Portal vein tumor thrombus, *NIACE* the number of nodules, the infiltrating nature of the HCC, α-fetoprotein serum level, Child–Pugh score, and Eastern Cooperative Oncology Group Performance Status grade, *PROSASH* PRediction Of Survival in Advanced Sorafenib-treated HCC, *ILIS* the Integrated Liver Inflammatory Score, *SPSM* Sorafenib-treated Prognostic Scoring Models, *IBSs-SII* Inflammation-based scores- systemic immune-inflammation index, *3-GPI* a 3-group prognostic index, *OTE* Off-target effects, *SAP* Sorafenib Advanced HCC Prognosis, *LMR-N* Lymphocyte to monocyte ratio-nomogram, *RD-CLIP* baseline Red cell distribution width-Cancer of Liver Italian Program, *NBBM* Novel biomarker-based model, *GPS* The Glasgow Prognostic Score, *PROSASH-II* PRediction Of Survival in Advanced Sorafenib-treated HCC-II, *NEXT* Survival after Stopping Nexavar Treatment, *BCP* the Baseline C-reactive protein Prognostic, *ACIKCI-N* Adjuvant Cytokine-Induced Killer Cell Immunotherapy-nomogram, *FOLFOX4-N* FOLFOX4-nomogram, *GPS-EP* portal thrombosis and GPS within an extended score, *JRC* Proposal of Japan Red Cross score, *9-MRG* Nine metabolism-related genes, *SCHCC* Sub-classification of Advanced-Stage Hepatocellular Carcinoma, *PBNC-N* Peripheral blood neutrophil count-nomogram, *7-IRGBM* Seven Immune-Related genes-based model, *IGPPM* ImmuneGene Pairs Prognostic Model, *8-IPSHCC* a Novel 8 Immune Gene Prognostic Signature, *10-IRGPM* Ten Immune-Related Genes Prognostic Model, *9-NIRPM* a novel 9 immune-related prognostic model

### Development of prognostic models

#### Research time and publication time

Among the 28 developed prognostic models, the earliest study was in 2000, and the most recent study was in 2017. The longest study interval was 11 years and the shortest was 2 years. The earliest articles reporting the development of these models were published in 2013; the year with the most such publications was 2017 (*n* = 9), followed by 2020 (*n* = 7).

#### Countries

Among the 28 prognostic models, six were developed based on The Cancer Genome Atlas (TCGA) and International Cancer Genome Consortium (ICGC) databases, and the other 22 models were mainly developed in South Korea (*n* = 5), France (*n* = 4), China (*n* = 4), the United Kingdom (*n* = 3), Italy (*n* = 3), Germany (*n* = 3), and Japan (*n* = 3), among which there were also multiple prognostic models jointly developed by multiple countries.

#### Intervention methods

The prognostic models we collected involved patients with HCC after receiving systemic treatment. The systemic treatment methods for HCC include targeted therapy (e.g., sorafenib, lenvatinib, regorafenib, cabozantinib, ramucirumab), immunotherapy (e.g., nivolumab and pembrolizumab), and other treatments (FOLFOX-4). Most of the 28 prognostic models were developed based on sorafenib treatment (*n* = 19). Other intervention methods included various undifferentiated treatments, including systemic therapy (*n* = 7), immunotherapy (*n* = 1) [47], and FOLFOX-4 (*n* = 1) [48].

#### Validation type

Newly developed prognostic models are always subject to internal validation to quantify their predictive ability on the same dataset. The most common internal validation methods include bootstrapping and cross-validation, but attention should be focused on the problem of overfitting. However, it is necessary to externally verify the prognostic model in multiple independent datasets, that is, to validate and even update the original model in different regions and backgrounds, and independent populations. Among the 28 prognostic models, 11 had undergone both internal and external validation, nine had only undergone internal validation, two had only undergone external validation, and the remaining six had not undergone any validation.

#### Sample size

In some articles, the research population was from the same study center, and the model was developed for these populations with or without internal validation. In other articles, the research populations from different study centers were divided into development and validation cohorts. Model development and internal validation were carried out in the development cohort, and model performance was reassessed in the validation cohort. For the 28 prognostic models, the average sample size of the development cohort was 373; the average sample size of the internal validation cohort was 402, and that of the external validation cohort was 308.

#### Clinical outcome

The most common clinical indicators for predicting the prognosis of patients with HCC after systemic treatment were OS and PFS. OS was defined as the time interval from the first clinical diagnosis of HCC to death, or last follow-up if death had not occurred. PFS was defined as the time interval from the beginning of systemic treatment to disease progression or death from any cause. In the 28 prognostic models, we mainly extracted OS to facilitate statistics.

#### Predictors

**Fig. 2:**
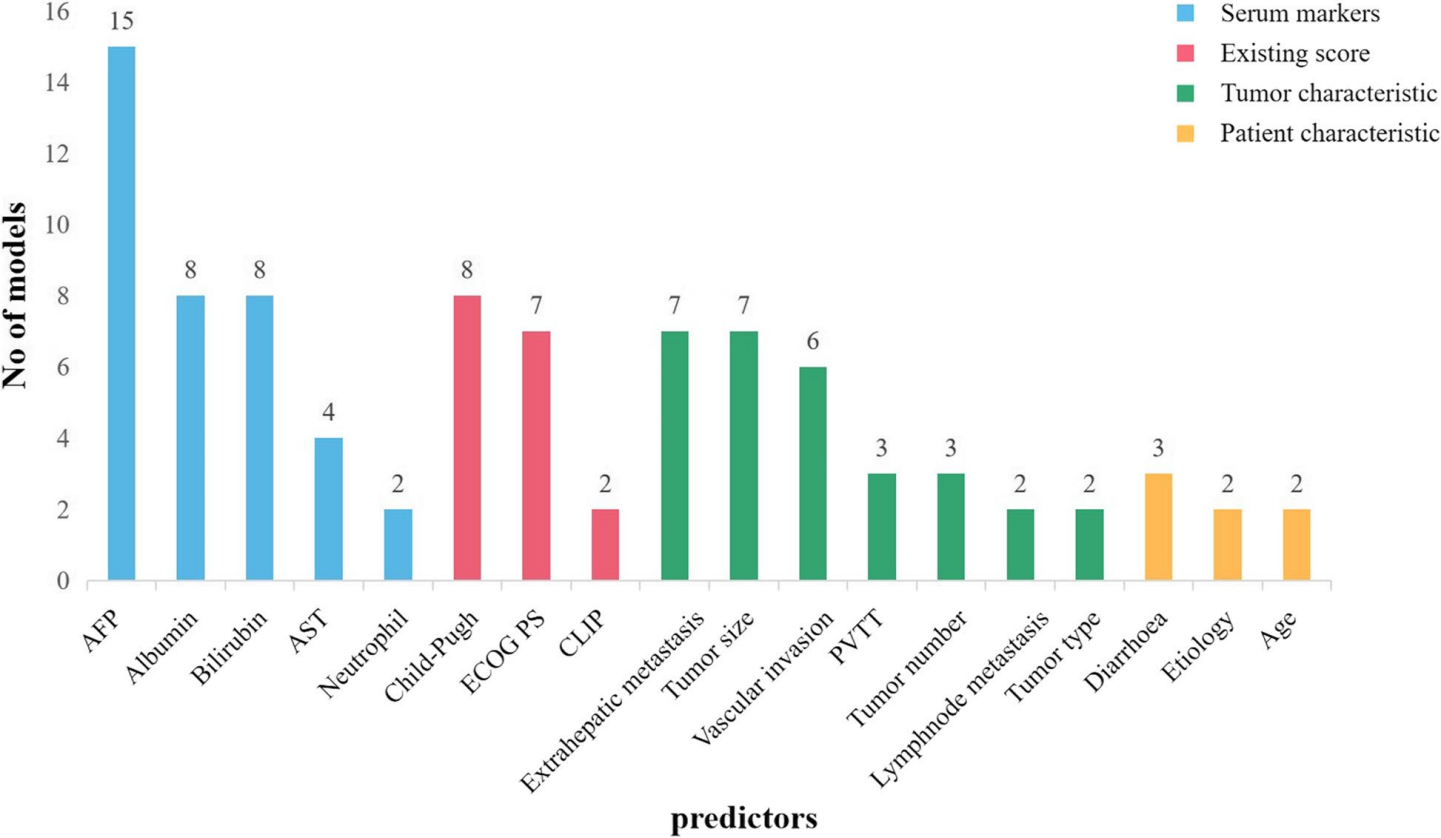
Predictors included in 23 prognostic models for HCC patients by category of predictor

Among the 28 prognostic models, five were based on TCGA and ICGC databases and used genes as predictors, and treatment was not limited to systemic treatment. These prognostic models were expressed in the form of equations (shown in Table 2); another prognostic model was also developed based on TCGA database, but its treatment was sorafenib. The predictors of the other 22 models were based on clinically accessible factors, including serum markers, existing scoring systems, tumor-related characteristics, and patient-related characteristics. The most commonly used predictors were AFP (*n* = 9), albumin (*n* = 8), bilirubin (*n* = 8), Child–Pugh class (*n* = 8), extrahepatic metastasis (*n* = 7), tumor size (*n* = 7), and vascular invasion (*n* = 6) (Fig. 2).

**Table 2:** Model equations of prognostic models for gene-related in hepatocellular carcinoma

Author	Prediction model	Model equation
Tang C, 2020 [50]	9-MRG	(0.0193 × RRM2) + (0.0068 × DTYMK) + (0.0003 × LPCAT1) + (0.0013 × LCAT) + (0.0087 × TXNRD1) + (0.0035 × G6PD) + (0.0012 × PTGES) + (0.0508 × ENTPD2) + (0.0729 × UCK2)
Liu T, 2020 [52]	7-GBM	(BIRC5 × 0.0238) + (FOS × 0.0055) + (DKK1 × 0.0085) + (FGF13 × 0.3432) + (IL11 × 0.0135) + ( IL17D × 0.0878) + (SPP1 × 0.0003)
Xu D, 2020 [54]	8-IPSHCC	(0.00109 × CKLF) + (0.23932 × IL12A) + (0.00067 × CCL20) + (0.01209 × PRELID1) + (0.09808 × FYN) + (0.08045 × GLMN) + (0.07259 × ACVR2A) + (0.00434 × CD7)
Wang WJ, 2020 [55]	10-IRGPM	( BIRC5 × 0.02296) + (CSPG5 × 0.33178) + ( IL-11 × 0.01577) + (FABP6 × 0.07392) + (FIGNL2 × 0.44366) + (GAL × 0.18222) + (IL17D × 0.08771) + (MAPT × 0.27133) + ( SPP1 × 0.00015) + (STC2 × 0.02978)
Wang Z, 2020 [56]	9-NIRPM	(0.2940 × ANGPT1) + (0.1753 × MAPT) + (0.1066 × DCK) + (0.0706 × SEMA3F) + (0.0703 × IL17D) + (0.0311 × HSPA4) + (0.0204 × RBP2) + (0.0084 × NDRG1) + (0.0052 × OSGIN1)

*9-MRG* Nine metabolism-related genes, *GBM* Seven Genes-based model, *IPSHCC* a Novel 8 Immune Gene Prognostic Signature, *10-IRGPM* Ten Immune-Related Genes Prognostic Model, *9-NIRPM* a novel 9 immune-related prognostic model;

#### Model performance

The most common indicators for evaluating the predictive performance of a prognostic model were discrimination and calibration. Discrimination refers to the predictive ability to distinguish whether an individual will have an outcome event, that is, it can correctly distinguish patients with different risks of prognosis. The most commonly used indicator was the area under the ROC curve, also termed the C statistic. A larger value indicated better discriminative ability of the prediction model, and was between 0.5 and 1. Among the articles on the 28 prognostic models, 24 calculated the model’s C statistic. Calibration is the accuracy of the predictive model for predicting the probability that an individual will have an outcome event, which refers to the consistency between the model’s predicted risk and the actual risk, so it is also termed consistency. In practical applications, the calibration chart can visually display the relationship between the predicted risk and the actual risk, or calculate the Hosmer–Lemeshow goodness-of-fit test. Most of the 28 prognostic models did not present a calibration chart, and only four articles described the calibration chart.

### External validation of prognostic models

Thirty-two prognostic models were externally validated. Most of these models were originally developed for HCC prognosis prediction. Only four models were developed specifically for the prognosis prediction of patients with HCC with systemic treatment. They were Prediction Of Survival in Advanced Sorafenib-treated Hepatocellular carcinoma (PROSASH) [17], PROSASH-II [18], Sorafenib Advanced HCC Prognosis (SAP) [40] and NIACE [33]). The data extraction form for the external validation is included in Table 3.

**Table 3:** Data from 14 articles that externally validated 32 existing prognostic models for HCC patients undergoing systemic therapy

Model	c-statistics	95%CI lower	95%CI upper	Outcome	Type of cohort(N)	events(n)
PROSASH-II	0.65	NA	NA	NA	Training (615)	559
	0.68	0.65	0.72	OS	Validation (290)	273
	0.63	0.6	0.65	OS	Validation (552)	NA
PROSASH	0.72	0.69	0.75	OS	Training (500)	NA
	0.7	0.67	0.73	OS	Validation (421)	
	0.71	0.69	0.73	OS	All(500 + 421)	
	0.63	0.6	0.66	OS	Validation (438)	NA
ALBI	0.6	NA	NA	NA	Validation (905)	NA
	0.595	0.566	0.625	OS	Validation (468)	NA
	0.56	0.51	0.6	OS	Validation (681)	NA
	0.59	NA	NA	NA	Validation (615)	559
	0.62	0.58	0.65	NA	Validation (290)	273
	0.53	0.51	0.56	OS	Validation (552)	NA
	0.638	0.599	0.676	OS	Validation (900)	598
	0.748	0.732	0.763	OS	Validation (3182)	NA
Child–Pugh	0.61	NA	NA	NA	Validation (905)	NA
	0.53	NA	NA	NA	Validation (615)	559
	0.58	0.55	0.61	OS	Validation (290)	273
	0.584	0.539	0.629	FFS	Validation (201)	155
	0.592	0.547	0.637	OS	Validation (201)	155
	0.52	0.51	0.54	OS	Validation (552)	NA
	0.638	0.598	0.678	OS	Validation (900)	598
BCLC	0.637	0.58	0.695	OS	Validation (435)	NA
	0.55	0.528	0.571	OS	Validation (468)	NA
	0.56	0.52	0.6	OS	Validation (681)	NA
	0.54	NA	NA	NA	Validation (615)	559
	0.57	NA	NA	NA	Validation (290)	273
	0.57	0.55	0.6	OS	Validation (552)	NA
	0.65	NA	NA	OS	Validation (3628)	NA
	0.64	NA	NA	OS	Validation (1555)	NA
	0.73	NA	NA	OS	Validation (2651)	NA
	0.739	0.709	0.769	OS	Validation (1013)	NA
	0.678	0.559	0.796	OS	Validation (108)	NA
	0.665	0.653	0.678	OS	Validation (1969)	NA
	0.71	0.67	0.74	OS	Validation (904)	508
	0.727	0.692	0.762	OS	Validation (900)	598
	0.807	0.773	0.824	OS	Validation (1188)	652
HAP	0.653	0.624	0.681	OS	Validation (468)	NA
	0.6	NA	NA	NA	Validation (615)	559
	0.67	0.64	0.7	OS	Validation (290)	273
	0.59	0.56	0.62	OS	Validation (552)	NA
SAP	0.64	0.614	0.667	OS	Validation (468)	NA
	0.6	NA	NA	NA	Validation (615)	559
	0.69	0.66	0.72	OS	Validation (290)	273
	0.58	0.55	0.61	OS	Validation (552)	NA
JIS	0.691	0.638	0.744	OS	Validation (435)	NA
	0.55	NA	NA	OS	Validation (615)	559
	0.59	0.55	0.62	OS	Validation (290)	273
	0.67	NA	NA	OS	Validation (3628)	NA
	0.67	NA	NA	OS	Validation (1555)	NA
	0.7	NA	NA	OS	Validation (2651)	NA
	0.753	0.748	0.771	OS	Validation (1969)	NA
	0.7	0.66	0.73	OS	Validation (477)	295
	0.78	0.76	0.8	OS	Validation (904)	508
	0.798	0.774	0.823	OS	Validation (1188)	652
CLIP	0.542	0.485	0.599	OS	Validation (435)	NA
	0.66	0.6	0.72	OS	Validation (681)	NA
	0.642	0.604	0.68	FFS	Validation (201)	155
	0.642	0.605	0.679	OS	Validation (201)	155
	0.69	NA	NA	OS	Validation (3628)	NA
	0.68	NA	NA	OS	Validation (1555)	NA
	0.75	NA	NA	OS	Validation (2651)	NA
	0.76	0.748	0.771	OS	Validation (1969)	NA
	0.7	0.66	0.74	OS	Validation (477)	295
	0.75	0.73	0.77	OS	Validation (904)	508
	0.777	0.744	0.809	OS	Validation (900)	598
	0.75	0.727	0.774	OS	Validation (1188)	652
Okuda	0.632	0.577	0.687	OS	Validation (435)	NA
	0.64	0.6	0.69	OS	Validation (681)	NA
	0.639	0.601	0.676	FFS	Validation (201)	155
	0.68	0.641	0.718	OS	Validation (201)	155
	0.733	0.718	0.748	OS	Validation (1973)	NA
JRC	0.755	0.707	0.803	OS	Training (435)	NA
AJCC TNM7	0.56	0.5	0.62	OS	Validation (681)	NA
	0.755	0.724	0.785	OS	Validation (1013)	NA
	0.741	0.635	0.847	OS	Validation (108)	NA
	0.675	0.659	0.691	OS	Validation (1973)	NA
SCHCC	0.818	NA	NA	OS	Training (612)	NA
MESIAH	0.77	0.74	0.8	OS	Training (477)	295
	0.82	0.8	0.83	OS	Validation (904)	508
	0.69	NA	NA	OS	Validation (3628)	NA
	0.69	NA	NA	OS	Validation (1555)	NA
	0.77	NA	NA	OS	Validation (2651)	NA
	0.835	0.81	0.861	OS	Validation (1013)	NA
	0.785	0.687	0.884	OS	Validation (108)	NA
	0.792	0.782	0.803	OS	Validation (1969)	NA
Modified BCLC	0.66	NA	NA	OS	Validation (3628)	NA
	0.66	NA	NA	OS	Validation (1555)	NA
	0.75	NA	NA	OS	Validation (2651)	NA
HKLC	0.68	NA	NA	OS	Validation (3628)	NA
	0.68	NA	NA	OS	Validation (1555)	NA
	0.75	NA	NA	OS	Validation (2651)	NA
ITA.LA.CA	0.57	0.54	0.59	OS	Validation (552)	NA
	0.72	NA	NA	OS	Training (3628)	NA
	0.71	NA	NA	OS	Validation (1555)	NA
	0.78	NA	NA	OS	Validation (2651)	NA
Tokyo score	0.716	0.703	0.729	OS	Validation (1969)	NA
	0.702	0.685	0.719	OS	Validation (1973)	NA
	0.767	0.742	0.793	OS	Validation (1188)	652
APRI	0.569	0.53	0.608	OS	Validation (900)	598
PALBI	0.67	0.632	0.707	OS	Validation (900)	598
	0.78	0.765	0.794	OS	Validation (3182)	NA
FIB-4 score	0.540	0.5	0.579	OS	Validation (900)	598
MELD	0.591	0.551	0.631	OS	Validation (900)	598
	0.664	0.647	0.68	OS	Validation (3182)	NA
CTP	0.742	0.727	0.757	OS	Validation (3182)	NA
CP-based BCLC	0.757	0.741	0.774	OS	Validation (1973)	NA
ALBI-based BCLC	0.76	0.743	0.776	OS	Validation (1973)	NA
CP-CLIP	0.785	0.769	0.802	OS	Validation (1973)	NA
ALBI-CLIP	0.789	0.772	0.806	OS	Validation (1973)	NA
ALBI-based JIS (ALBI-T)	0.74	0.723	0.757	OS	Validation (1973)	NA
LCSGJ TNM	0.686	0.67	0.703	OS	Validation (1973)	NA
CUPI	0.708	0.696	0.72	OS	Validation (1973)	NA
	0.701	0.679	0.722	OS	Validation (1188)	652
CP-based JIS	0.734	0.717	0.751	OS	Validation (1973)	NA
GRETCH	0.688	0.664	0.713	OS	Validation (1188)	652

*AJCC TNM* American Joint Committee on Cancer Tumour Node Metastasis, *FFS* Failure-free survival, *ALBI* Albumin-bilirubin, *BCLC* Barcelona Clinic Liver Cancer, *CLIP* Cancer of the Liver Italian Program score, *HAP* Hepatoma arterial-embolization prognostic score, *JIS* Japan Integrated Staging score, *JRC* Japan Red Cross score, *MESIAH* Model to Estimate Survival in Ambulatory HCC patients, *PROSASH* Prediction Of Survival in Advanced Sorafenib-treated HCC, *SAP* Sorafenib Advanced HCC Prognostic score, *APRI* Aspartate aminotransferase-to-platelet ratio index, *PALBI* Platelet-albumin-bilirubin index, *FIB-4 score* Fibrosis-4, *MELD* Model for end-stage liver disease score, *CTP* Child–Turcotte–Pugh, *CUPI* Chinese University Prognostic Index, *LCSGJ* Liver Cancer Study Group of Japan, *GRETCH* Groupe d’Etude du Treatment du Carcinome H′epatocellulaire

### Risk of bias assessment

We used PROBAST [14, 15] to assess the risk of bias of all studies in the development of prognostic models (except for the five genetic prognostic models). Unfortunately, all models had a high risk of bias, which may limit their application in clinical practice.

Among the remaining 23 articles of prognostic model development, 15 had a high risk of bias in the participant domain, which indicates that the study’s participants may not be representative of the model’s target population. These studies usually collect existing data retrospectively, and the study participants’ inclusion and exclusion criteria are inappropriate. In addition, four articles had low risk of bias, and four articles had unclear risk of bias in this domain. In the predictor domain, most studies (*n* = 15) had a low risk of bias. The researchers used the same method to define and measure predictors. Predictors are assessed without knowing the status of the clinical outcome. When the predictive model is used, information about all predictors in the model can be obtained. In addition, six and two articles had unclear and high risk of bias, respectively. In terms of outcomes, most studies (*n* = 21) had a low risk of bias, as most of their clinical outcomes were OS and PFS, which are considered superior outcome indicators in the guidelines. It is an objective standard, excluding predictors, and all participants used similar methods to define and determine clinical outcomes. Outcomes are also determined without knowing the predictors’ information, and the interval between predictor measurement and outcome determination was appropriate. In addition, two articles had unclear risk of bias in this domain.

**Fig. 3:**
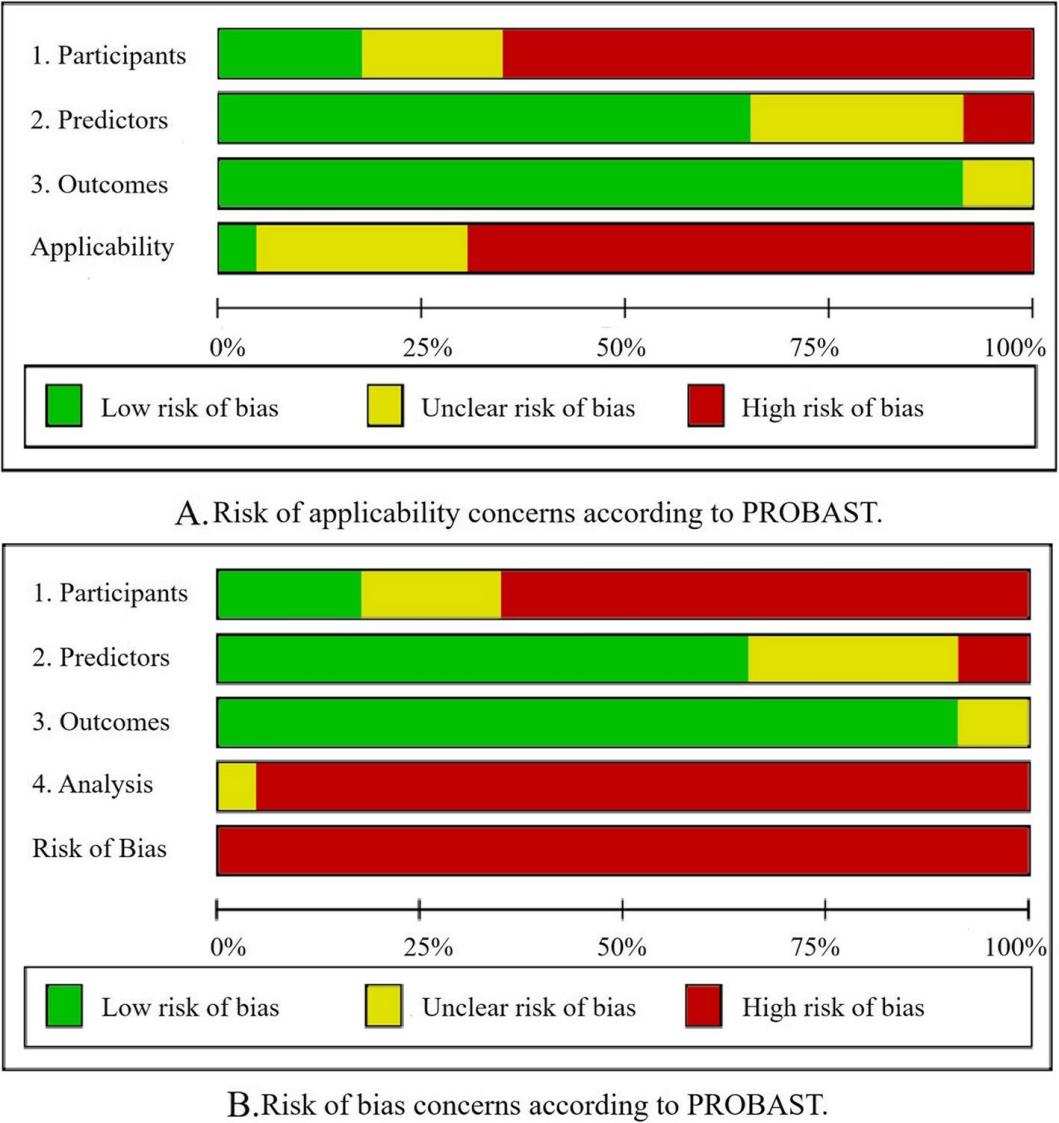
Risk of applicability and bias concerns according to PROBAST

The applicability assessment of the participants, predictors, and outcomes of the 23 studies mainly depended on whether these three domains matched the research questions of the systematic review. In general, 16 studies had poor applicability, six studies had unclear applicability, and one study had good applicability. The prognostic model with good applicability was the NBBM model [43]. The results of risk of applicability concerns according to PROBAST are shown in Fig. 3A.

All studies had a high risk of bias in the statistical analysis domain. The problems are as follows: small sample size and greater risk of overfitting; the continuous predictor was converted into categorical variables; some participants were deleted during data analysis; missing values were not properly handled; univariate analysis was used to select predictors and include them in a multivariate model; complex issues (e.g., missing data, competitive risk data, sampling of control participants) were not considered; internal validation was not performed, resulting in overfitting and optimistic bias in model performance; the predictors and regression coefficients in the final model did not match the results reported by the multivariate analysis. Due to the high risk in the statistical analysis domain, all models had high overall risk of bias (Fig. 3B).

## Discussion

We analyzed 28 articles describing 28 developed models for predicting the prognosis of patients with HCC with systemic treatment, and 14 articles that performed external validation of 32 traditional or classic models for patients with HCC receiving systemic treatment. The development and validation of these models will aid the identification of patients with HCC who may benefit from systemic therapy, and guide treatment. Assessment of the performance of 14 of the 28 developed models (C statistics and 95% CI) showed that they had good predictive performance. However, due to the inappropriate design of the participants, predictors, outcomes, and the most important statistical analysis methods, these models had high risk.

### Principal findings in context

Among the prognostic models developed, less than a quarter were developed based on TCGA and ICGC databases, and their predictors were genes. Five models were developed with immune-related genes (IGR) as predictors. Liu et al. included seven IGR [52], Xu et al. used eight IGR [54], Wang et al. included nine IGR [56], Wang et al. included 10 IGR [55], and Huo et al. included 45 IGR [53]. These authors established immune-based prognosis models for HCC, which not only provided new potential prognostic biomarkers and therapeutic targets, but also provided clinical data support for the theoretical basis of HCC immunotherapy. Tang et al. constructed a prognostic model based on nine metabolism-related genes (MRG) [50]. Twenty-two non-gene prognosis models were developed mainly in Asian countries such as South Korea, China, and Japan, while the rest were developed in Western countries. The risk of HCC varies according to geographic region, gender, age, and impaired liver function. The incidence of HCC in Asia is high, and there are strong diagnosis and treatment needs [57]. Globally, the leading cause of HCC is HBV infection, mainly in Asia and sub-Saharan Africa. In Western countries and in Japan, the main causes of HCC are HCV infection and nonalcoholic fatty liver disease (NAFLD) [58]. Most of the current predictive models for patients with HCC with systemic treatment were developed in a single country or single research center, without external validation in other countries or centers, requiring further external validation to assess their predictive performance.

### Statistical methodological flaws

Our systematic review reveals some statistical methodological pitfalls in the models’ development, rendering these models at high risk of biased assessment. Five-sevenths of the models were internally validated, 50% were externally validated, and 25% were not validated. When the predictive performance of a model is quantified with modeled data, the estimations made are more optimistic, which can cause overfitting. Therefore, the model should undergo internal validation, such as bootstrapping and cross-validation. In addition, for models that have experienced optimistic bias, there is a need to adjust or reduce the estimated performance of the model prediction and readjust the predictors’ regression coefficients in the final model, although this is done for few models [59]. To generalize a model in different populations and areas, it is externally validated to assess the predictive performance of the existing model. Some classical staging systems for HCC have existed for many years and can be externally validated and updated for a more suitable prognosis model.

A key factor of our systematic review is the discrimination and calibration of the prognostic models [60]. The most commonly used and widely cited discrimination indicator is the concordance index (c-index or C statistic). Calibration is commonly represented in the form of a calibration plot and the Hosmer–Lemeshow goodness-of-fit test [15, 60]. Poor calibration may be due to the direct deletion of missing data, or the conversion of continuous variables into categorical variables. The model’s discrimination and calibration should be evaluated to explore the overall scope of the model’s predictive risk and the full assessment of the predicted performance. If they are not evaluated, the study faces a certain risk of bias, and the model may be unable to make accurate risk predictions for individuals.

Another key factor in our systematic review is the clinical application value of the model. In addition to assessing the risk of bias in PROBAST, we evaluated the applicability of the model to the intended target population and clinical environment. When the participants, predictors, or outcomes are different from these elements required in the model, whether the original study also applies to the question of systematic review research should be determined [10, 11]. In the 23 developed prognostic models for sorafenib, 16 were less applicable, six had unclear applicability, and one was more applicable, and was the NBBM model [43]. In addition, whether prognostic models are beneficial to clinical practice requires decision analysis and model presentation [61]. The most commonly used decision analysis tools in clinical practice are scoring systems, decision trees, nomograms, and full equations. Of the 28 developed models, one-seventh of the models had no model presentation, 15 were layered with scoring systems, six were represented by nomograms, and six were expressed in full equations. Decision analysis tools make models more convenient for clinical applications.

### Clinical application

The most commonly used predictors for developing prognostic models were AFP, albumin, bilirubin, Child–Pugh class, liver metastasis, tumor size, and vascular invasion. These predictors are important factors in the natural process of disease, and some are biomarkers of disease severity. One advantage of these predictors is that they are easy to measure, and serum and imaging examination is a routine examination item for clinical hospitalization and is easy to obtain. Another advantage of these predictors is low measurement risk. Blood samples and imaging tests inflict minor damage on the patient and have less misclassification. Finally, these predictors have been identified as individual prognostic factors in patients with HCC, especially AFP, the main biomarker of HCC diagnosis, and their changes reflect the disease severity [62, 63]. After systemic treatment, the prognosis of patients with HCC can be predicted based on the model of these clinical indicators, and more appropriate treatment methods can be selected. However, these newly developed models require greater sample sizes for further validation to promote their application and to optimize and update the original model.

In view of the better effect of systemic therapy in advanced HCC and the occurrence of adverse reactions, clinicians need to consider the advantages and disadvantages of systemic treatment. There are numerous studies for the external validation of the original classical models. BCLC, CLIP, JIS, ALBI, and Child–Pugh class are the most validated prognosis models. Although each staging system can predict and layer the prognosis of patients, some staging systems may not be suitable for patients with HCC who receive systemic treatment. BCLC is the most commonly used staging system in Western countries, incorporating performance status (PS), tumor-related variables (tumor size and number, liver metastasis, vein invasion), and liver function (Child–Pugh). BCLC grades the prognosis for patients with cirrhosis and curative HCC well, but the vast majority of patients with HCC receiving systemic treatment are in the BCLC C stage, which includes PS scores of 1–2, vascular invasion, extrahepatic metastasis, and Child–Pugh A/B. Therefore, it is not suitable for stratifying patients with HCC treated with systemic treatment and has limited prognostic effect on advanced HCC treated with systemic treatment. CLIP is one of the most commonly used staging systems, combining liver function (Child–Pugh score) with tumor-related characteristics (tumor size and morphology, portal vein tumor thrombus, AFP). It is commonly used for evaluating OS in patients with HCC. CLIP scoring classifies the majority of patients with medium-stage unresectable HCC. This indicates that CLIP has low predictive effects for patients with HCC who receive systemic treatment. This may be due to the lack of evaluation of PS in the scoring system, which is associated with the prognosis of HCC survival and is one of the main conditions of clinical trials for systematic therapy. In contrast, Asian researchers favor JIS more, and it includes tumor-related characteristics (tumor size and number, vascular invasion) and liver function (Child–Pugh score). When the model was evaluated in patients with HCC receiving systemic treatment only, its predictive effectiveness was reduced. JIS was unable to properly stratify patients with advanced HCC to assess prognosis, which is similar to the two staging systems mentioned above. ALBI only includes albumin and bilirubin, two indicators of liver function, which can reduce human subjectivity because of objective laboratory indicators. Compared to ALBI, the Child–Pugh score includes more subjective indicators (hepatic encephalopathy, ascites, bilirubin, albumin, prothrombin time). At present, most clinical trials of advanced HCC include patients with Child–Pugh A. Although these patients have better liver function, patients in the high-risk group have shorter medium OS and it is more difficult for them to benefit from systemic treatment. Accordingly, they should consider the best support treatment. Most of these models are not specifically designed for patients with HCC treated with systemic drugs, so they have low predictive performance and require the development of new models or updating of existing models for more precise clinical practice.

An important step of predictive models for clinical practice is to conduct external validation in populations from different clinical backgrounds, which can select predictive models with better performance through discrimination and calibration. Several external validations of prognostic models have been developed specifically for systemic therapy. PROSASH is a statistical model developed by Berhane, predicting average survival to assist patient consultation and trial design [17]. Subsequently, Labeur updated PROSASH by incorporating fewer subjective predictors and more objective predictors to develop PROSASH-II. It was superior to other models and provided risk stratification and individual survival prediction for sorafenib-treated patients with HCC [18]. Edeline et al. developed and validated the SAP model, which facilitates clinical decision-making and prognosis stratification [40]. The Hepatoma Arterial embolization Prognostic (HAP) model was originally designed for patients with HCC treated with TACE, but showed better discrimination in sorafenib-treated patients with HCC. It is recommended for evaluating the curative effect of systemic drug treatment in patients with HCC [64].

### Recommendations and policy implications

For the pitfalls of the statistical methods described above, broadly accepted recommendations are to take these factors into account in the model development process to improve the predictive ability. First, in model development, internal validation should be used to prevent overfitting, and shrinkage technology should be used to adjust model performance. Second, the prognostic model’s performance (i.e., discrimination and calibration) should be reported in a timely manner. If the prognosis model has poor consistency, it should be updated in a timely manner. Third, missing data should be handled by multiple imputation instead of being deleted directly. Fourth, continuous variables should not be converted directly into categorical variables, and the non-linear relationship between predictors and outcomes should be examined by fractional polynomials or restricted cubic splines. Finally, existing models should be externally validated in other countries or centers to test their predictive capacity and promote clinical practice.

### Strengths and limitations of the study

The main strength of our study is that it provides an overall map of the prognosis models for predicting the clinical outcomes in patients with HCC who receive systemic treatment. We describe the developed models and document the performance of existing models based on external validation in detail. In addition, we assessed the developed models’ risk of bias with the PROBAST tool.

The limitation is that there are major differences in the study population, treatment measures, statistical methods, and the number of external validations. The calibration cannot be calculated by meta-analysis due to the poor heterogeneity.

## Conclusions

We summarize the multivariate prognosis models for predicting clinical outcomes in patients with HCC with systemic treatment. Several models have been developed, and several classical models have been validated externally, so choosing the appropriate prognosis model is challenging for doctors. Future studies should focus on updating existing prognosis models by adjusting predictors to improve performance and promoting their clinical practice through external validation.

## Supplementary Information

**Additional file 1: Table S1.** Key items for framing aim, search strategy, and study inclusion and exclusion criteria for systematic review, following PICOTS guidance.

## Data Availability

All data generated or analysed during this study are included in this published article (and its supplementary information files).
