# Supplementary material for: Prognostic models for outcome prediction in patients with advanced hepatocellular carcinoma treated by systemic therapy: a systematic review and critical appraisal
Source: BMC Cancer. 2022 Jul 9;22:750. doi: 10.1186/s12885-022-09841-5 (PMC9270753; doi:10.1186/s12885-022-09841-5)
Supplement: Supplementary file 1 — Additional file 1: Table S1. Key items for framing aim, search strategy, and study inclusion and exclusion criteria for systematic review, following PICOTS guidance. [file 12885_2022_9841_MOESM1_ESM.docx]

Table- S1. Key items for framing aim, search strategy, and study inclusion and exclusion criteria for systematic review, following PICOTS guidance.

| Item | Comments |
| --- | --- |
| Population | Patients diagnosed as HCC receiving systemic treatment |
| Intervention | Any prognostic model to predict any possible clinical outcome in HCC patients |
| Comparator | Not applicable |
| Outcomes | Any clinical outcome reported by prognostic models |
| Timing | Predictors measured at any timepoint in clinical course of HCC and preceding outcome; outcome measured in short term or long term without applying any specific limitation in prediction horizon |
| Setting | To distinguish HCC patients with poor prognosis , or to aid decision making in acute care and treatment planning in long term |

HCC=hepatocellular carcinoma
